# Social perceptions of seafarers and the sustainable development of the maritime workforce: a questionnaire survey in South Korea

**DOI:** 10.3389/fpubh.2026.1927794

**Published:** 2026-09-21

**Authors:** Mingfei Ma, Jiakai Ma, Junghwan Choi, Byeongha Lee

**Affiliations:** 1 Law School, Dalian Maritime University, Dalian, China; 2 Korea Institute of Maritime and Fisheries Technology, Busan, Republic of Korea

**Keywords:** occupational attractiveness, seafarers, social perception, social policy, sustainable workforce, wellbeing

## Abstract

This study examines social perceptions of the seafaring profession and explores how these perceptions influence occupational attractiveness and the sustainable development of the maritime workforce. A questionnaire survey was conducted among students, the general public, and active seafarers, examining multidimensional factors including occupational image, knowledge level, working conditions, career choice and recommendation intentions, and perceived social value. The collected data were analyzed using descriptive statistics and one-way analysis of variance (ANOVA) to identify differences in perceptions among the respondent groups. The findings indicate that, despite the critical role of seafarers in global shipping, maritime logistics, and national maritime security, their societal value and professional recognition remain low. Significant differences were identified across respondent groups. Students and members of the general public tended to overestimate the occupational image, whereas active seafarers reported negative assessments of working conditions and career sustainability. The study suggest that limited public recognition and unfavorable perceptions of employment conditions may reduce the attractiveness of seafaring careers and pose challenges to the sustainable development of the maritime workforce. The study highlights the need for evidence-based social policies, public awareness campaigns, and institutional interventions to strengthen occupational recognition, improve workforce wellbeing, and support the long-term sustainability of the maritime industry.

## 1 Introduction

The shipping industry is a core pillar of the national economy and logistics system, structured around three essential elements: capital, vessels, and human resources in the form of seafarers (1). Among these, seafarers constitute an irreplaceable workforce that potentially associated with the safety and efficiency of ship operations and, ultimately, the sustainability of the shipping industry. Far beyond being merely technical personnel responsible for vessel navigation, seafarers undertake complex and high-level responsibilities, including responding to maritime emergencies, ensuring the safe transportation of cargo, and complying with international maritime regulations. Seafarers serve as key contributors to the stability of national logistics systems and the competitiveness of the shipping industry (2).

Despite this critical importance, seafaring is characterized by working conditions that are distinctly different from those of other occupations. These include prolonged separation from family, confinement within limited spaces, substantial physical and psychological demands, and persistent social isolation. In practice, seafarers often spend several months, and in some cases more than a year, living and working in the closed environment of a vessel, during which they are repeatedly exposed to psychological stress, physical fatigue, and the disruption of social relationships (3). Nevertheless, the social value and professional role of seafarers have not been adequately recognized (4). Moreover, in terms of welfare provisions and employment stability, the seafaring profession remains in a relatively vulnerable position compared to other industries. While some shipping companies have made voluntary efforts to improve welfare systems, enhance employment stability, and ensure adequate rest periods between embarkation and disembarkation in consideration of the occupational characteristics of seafarers, such initiatives remain largely confined to the corporate level (4). Institutional support at the national level, as well as broader social recognition and respect for the seafaring profession, remain insufficient (5). Low social perception of seafaring as a career may reduce occupational attractiveness, discourage younger generations from entering the profession, and undermine long-term career retention. These trends contribute to structural challenges such as workforce aging and shortages, which may ultimately weaken the competitiveness and sustainability of the shipping industry.

Social perception refers to the collectively shared attitudes, values, and common perspectives that members of society hold toward a particular phenomenon (6). In the context of occupational groups, social perception plays an important role in shaping occupational image, career attractiveness, and long-term workforce sustainability. In particular, negative social perceptions toward the seafaring profession may contribute to declining interest among younger generations, reduced occupational continuity, and workforce aging. These trends may ultimately weaken the competitiveness and sustainability of the shipping industry. Nevertheless, previous studies have mainly focused on seafarers' working conditions, welfare systems, and labor shortages, while relatively limited attention has been paid to the broader structure of social perceptions toward the seafaring profession and the factors influencing such perceptions.

Against this background, this study aims to empirically examine the level and structural characteristics of social perceptions toward the seafaring profession. To achieve this objective, the study employs a questionnaire-based quantitative research design targeting multiple social groups, including members of the general public, students of maritime education institutions, and active seafarers. The survey instrument initially consisted of 11 items measured on a five-point Likert scale, covering diverse perceptual dimensions such as occupational image, willingness to recommend seafaring as a career, employment stability, working conditions, welfare systems, and social recognition. A total of 491 valid responses were collected through convenience and snowball sampling methods, and the data were analyzed using descriptive statistics and one-way analysis of variance (ANOVA) with IBM SPSS Statistics version 22 (IBM Corp., Armonk, NY, USA) in order to identify overall perceptual tendencies and statistically significant inter-group differences.

Based on this empirical framework, the study systematically identifies both general patterns and group-specific disparities in perceptions of the seafaring profession. In doing so, it contributes to the existing literature by expanding the discussion of seafarer-related issues beyond traditional concerns such as labor shortages and working conditions toward the broader dimension of social perception. The findings also provide empirical evidence supporting the importance of social perception as a factor related to occupational attractiveness and the long-term sustainability of the maritime workforce. Furthermore, the study empirically demonstrates that, despite the fact that seafaring is a core professional occupation underpinning the national shipping industry and maritime security, its societal value and professional significance have not been sufficiently recognized by the public. The research therefore provides important academic and policy implications regarding the necessity of improving social recognition of the seafaring profession and establishing institutional support mechanisms. Ultimately, this study is expected to provide a foundational basis for developing seafarer-based public policies, awareness campaigns, and sustainable human resource strategies for the shipping industry.

## 2 The seafaring profession: occupational context and social perceptions

### 2.1 Theoretical framework: Social Cognitive Career Theory (SCCT)

Social Cognitive Career Theory (SCCT), developed by Lent et al. (7), provides a comprehensive theoretical framework for explaining how individuals develop career interests, make occupational choices, and maintain career persistence. Building upon Bandura's Social Cognitive Theory, SCCT proposes that career development is shaped through the interaction of three core components: self-efficacy, outcome expectations, and personal goals, together with contextual supports and barriers (7).

According to SCCT, career decisions are influenced not only by individuals' abilities and interests but also by environmental and social factors surrounding a particular occupation. Social evaluation, occupational image, family expectations, media representation, and institutional support constitute important contextual factors that influence individuals' perceptions of career attractiveness. Positive social recognition strengthens favorable outcome expectations and increases the likelihood that individuals will enter and remain in a profession, whereas negative social perceptions may discourage career entry and reduce long-term occupational commitment. This theoretical perspective is particularly relevant to the seafaring profession. Unlike many land-based occupations, seafaring is characterized by prolonged separation from family, social isolation, demanding working conditions, and relatively limited public understanding of its professional value (8). Consequently, public perceptions of seafaring are likely to influence not only the occupational image of the profession but also individuals' willingness to pursue maritime careers. When seafaring is perceived as socially undesirable or lacking professional recognition, potential entrants may regard the occupation as less attractive despite its economic and professional merits. Furthermore, SCCT suggests that career sustainability depends not only on initial career choice but also on continued commitment to the profession. In the maritime sector, negative social perceptions may reduce occupational pride among active seafarers, weaken long-term career intentions, and contribute to workforce aging and recruitment difficulties. These outcomes ultimately affect the sustainability of the maritime workforce and the long-term competitiveness of the shipping industry.

Based on this theoretical framework, the present study assumes that social perception represents an important contextual factor associated with occupational attractiveness and workforce sustainability. Accordingly, this study examines differences in perceptions of the seafaring profession among the general public, maritime students, and active seafarers, with the aim of providing empirical evidence for understanding how social perception may relate to the sustainable development of the maritime workforce.

### 2.2 Occupational distinctiveness of seafaring

Seafarers are characterized by prolonged separation from family and society (8, 37). This reflects the non-family-oriented and asocial nature of their work. They often experience a lack of stable organizational affiliation due to subordination to ship-specific command and control structures (9). Their labor capacity is continuously depleted by high-intensity physical and psychological demands (10). Moreover, seafarers face heightened vulnerability and isolation, as access to external assistance is severely limited in the event of accidents (11, 38). Owing to these combined characteristics, seafaring has traditionally been perceived as a difficult, hazardous, and socially undesirable occupation.

At the international level, the occupational distinctiveness of seafaring is also statistically substantiated. According to the BIMCO Seafarer Workforce Report, seafarers from Asia account for approximately 44% of the global seafaring workforce, with Asia functioning as the most significant labor-supplying region for the global shipping industry (12). However, the major Asian seafarer-supplying countries largely consist of developing economies, which tend to rely on foreign exchange earnings generated by supplying their national seafarers to vessels owned by foreign shipowners (4). This international division of labor constitutes a structural factor contributing to the relatively low level of social recognition and institutional protection afforded to Asian seafarers, despite the substantive role they perform within the global shipping industry (4).

Recent developments in vessel enlargement, automation, and communication technologies have led to assessments suggesting that the risks and isolation associated with maritime work have been mitigated (13). Nevertheless, seafarers are still required to respond autonomously to a wide range of hazards arising at sea, and technological advancement alone has inherent limitations in fully alleviating the fundamental isolation and heavy responsibility embedded in maritime labor (14). Despite this, such changes tend to foster social perceptions that underestimate the occupational distinctiveness of seafaring, as they fail to adequately reflect the actual conditions of seafarers' work.

In particular, the low social status accorded to the seafaring profession is closely associated with young people's reluctance to enter the occupation, a decline in occupational pride among active seafarers, and weakened intentions to pursue long term careers (15). These dynamics ultimately contribute to the aging of the seafaring workforce and to structural imbalances in the supply and demand of maritime labor. Based on of SCCT perspectives, these social perceptions function as contextual factors that influence occupational image, career attractiveness, and long-term occupational commitment. Consequently, negative perceptions of the seafaring profession may discourage career entry and reduce workforce retention, thereby affecting the long-term sustainability of the maritime workforce.

The challenges facing the seafaring profession cannot be explained solely by harsh working conditions (39). They should also be understood in terms of the interaction between occupational characteristics and the social environment surrounding the profession. Improving public recognition of the professional role and social value of seafarers is therefore essential not only for enhancing seafarer welfare and employment stability but also for strengthening occupational attractiveness and supporting the sustainable development of the maritime industry.

### 2.3 Current status and challenges of South Korean seafarers

The South Korean shipping industry constitutes a core national infrastructure sector, handling approximately 99.7 percent of the country's total import and export cargo volume and generating wide ranging spillover effects across related upstream and downstream industries, including shipbuilding, ports, and logistics (16). Beyond its economic significance, the industry also performs an essential strategic function in terms of national security by undertaking the transportation of military supplies and strategic materials in times of emergency (17). One of the key factors enabling these functions has been the long-term accumulation of high-quality human capital, particularly a well-trained and highly skilled seafaring workforce. South Korea has consistently produced competent maritime officers through a systematic maritime education and training system, and this pool of human resources has been highly regarded in the international shipping market (16).

Despite this industrial and strategic importance, however, the South Korean seafaring workforce has recently entered a phase of structural decline. During the period of rapid economic growth from the 1960s to the 1980s, seafarers played a pivotal role in earning foreign currency and advancing industrial development, functioning as a key driving force behind national economic growth. Overseas remittances from South Korean seafarers increased from USD 3.37 million in 1967 to USD 335.76 million in 1983, representing an increase of more than 100 fold over approximately 16 years (4). These remittances made a substantial contribution to foreign exchange acquisition and supported the country's broader industrialization efforts.

Nevertheless, in recent years, avoidance of maritime education and seafaring employment has become increasingly evident, particularly among the MZ generation. Younger cohorts tend to perceive seafaring as an occupation characterized by prolonged separation from family and society, irregular working schedules, high labor intensity, relatively low social status, and uncertainty regarding long term career continuity. These perceptions have contributed to a growing imbalance in the supply and demand of seafarers across the shipping industry. This trend should be understood not merely as an issue of individual occupational preference, but rather as a structural challenge—associated with the sustainability of the shipping industry (17). If such tendencies persist, shortages of skilled maritime professionals may become entrenched across the broader maritime sector, including shipbuilding, shipping, and port related industries, thereby potentially constraining South Korea's strategic objective of maintaining its position as a leading maritime nation.

In South Korea, maritime education and training institutions produce approximately 1,403 highly skilled seafarers annually (4). However, the number of nationally registered seafarers has continuously declined over the past 5 s, as shown in Table 1. This trend is attributed to factors such as the narrowing wage gap between shore-based and sea-based employment, dissatisfaction with existing welfare arrangements, prolonged separation from family, and the increasing societal emphasis on work-life balance.

**Table 1:** Trends in the number of employed Korean seafarers over the past 5 years (Unit: persons).

Category	2020	2021	2022	2023	2024
National seafarers	31,035	30,337	29,919	28,864	27,169
Overseas-Employed seafarers	2,530	2,173	1,948	1,723	1,562
Total	33,565	31,510	31,867	30,587	28,731

Source: Korea Seafarer's Statistical Yearbook (2025).

In response, domestic shipping companies have expanded the employment of foreign seafarers. As illustrated in Table 2, the number of foreign seafarers employed by South Korean shipping companies has steadily risen, compensating for the decline in national seafarers. While this approach may address short-term labor shortages, it raises concerns regarding the long-term accumulation of domestic maritime expertise and the intergenerational transmission of professional skills, both of which are essential for the competitiveness of the shipping industry.

**Table 2:** Employment status of foreign seafarers in South Korea (2017–2024; Unit: persons).

Year	Total	China	Indonesia	Vietnam	Myanmar	Philippines	Others
2017	25,301	1,669	8,275	4,720	4,512	5,903	222
2018	26,321	1,501	9,084	5,355	4,346	5,779	256
2019	26,331	1,304	9,498	5,452	4,306	5,557	214
2020	26,775	978	10,699	5,025	4,376	5,464	233
2021	27,333	625	11,166	4,405	4,653	5,880	604
2022	28,281	337	11,985	4,115	4,719	6,357	111
2023	30,436	218	13,107	4,322	5,025	6,878	151
2024	33,339	113	10,508	277	7,037	13,604	1,800

Source: Korea Seafarer's Statistical Yearbook (2025).

The challenges confronting South Korean seafarers should not be interpreted solely as a quantitative reduction in employment levels. Rather, they should be understood as a structural issue arising from the combined effects of diminished social recognition of the seafaring profession, employment instability, and inadequacies in welfare and career support systems. In order for the South Korean shipping industry to sustain its role as a core national and strategic sector, comprehensive policy responses are required. Such responses should include a reassessment of the value of seafarer human capital, improvements in the social recognition of seafaring occupations, and the reinforcement of stable employment and welfare frameworks.

### 2.4 Social perception issues regarding the seafaring profession

Social perceptions of the seafaring profession constitute a critical factor influencing labor inflow and occupational sustainability within the shipping industry, thereby exerting a substantial impact on its medium- to long-term development (15).

In particular, the portrayal of seafarers in mass media and news coverage tends to concentrate disproportionately on maritime accidents, occupational risks, prolonged absences, and unfavorable working conditions (18). This distorted perception further diminishes the social status of the seafaring profession. Distorted social evaluations of this nature not only erode seafarers' occupational pride but also operate as structural constraints on workforce recruitment and long-term retention (19).

Social support plays a critical role in buffering individuals from physical and psychological stress, while also facilitating adaptive coping strategies (20). In this respect, the seafaring profession is particularly sensitive to the level of social support it receives. However, the persistently low social evaluation of seafarers and the limited scope of societal support hinder the mitigation of occupational stress and are likely to diminish job satisfaction as well as long-term commitment to the profession.

In recent years, increasing attention has been directed toward the sustainability of the shipping industry, labor shortages, and the establishment of equitable and inclusive maritime working environments. Against this backdrop, the need to conduct multidimensional analyses of social perceptions of the seafaring profession and to develop corresponding institutional and policy responses has become increasingly evident. Unless efforts are undertaken to reconceptualize seafaring as a strategic and professional occupation underpinning national economic security and maritime safety, and to restore social respect and recognition for seafarers, the human resource base of the shipping industry is likely to weaken further over the long term.

## 3 Literature review

Previous studies on the seafaring profession can be broadly classified into two main strands: research focusing on the structural and environmental characteristics inherent in seafaring work, and studies examining the social issues that emerge as a consequence of these characteristics. These two strands are not analytically independent but are closely interconnected, and appear to be associated with the formation of social perceptions of seafaring and on the long-term sustainability of the occupation. In particular, as population aging among seafarers and declining entry by younger cohorts have become increasingly pronounced, there is a growing need to comprehensively examine both the occupational incentive structure of seafaring and its social perception.

The formulation of policies aimed at encouraging youth entry and securing a stable seafarer workforce must be grounded in an accurate understanding of the factors influencing career choice. Kim and Lee (21), based on interview surveys with prospective young seafarers, demonstrate that social perception plays a decisive role in career selection alongside economic compensation and employment stability. It may reflect that seafarer workforce policies should not be confined to improvements in working conditions or remuneration alone, but should be closely integrated with efforts to enhance the social image of the seafaring profession. Similarly, Kim and Lee (22) highlight that socially entrenched stereotypes portraying seafarers as transient workers, coupled with negative perceptions surrounding prolonged separation from family life, undermine the formation of social support for seafarers.

Meanwhile, Jo and D'Agostini (23), employing Q methodology to categorize public perceptions of seafaring, identifies a dominant negative image of the profession characterized by high risk, heavy workload, and low social recognition. This research implies that even when seafaring offers relatively high economic rewards, it may still be socially avoided due to persistent negative perceptions.

### 3.1 Occupational characteristics of seafarers

Seafaring is characterized by distinctive occupational features that clearly differentiate it from land-based professions, including prolonged periods of service at sea, the integration of living and working spaces, high-intensity physical and psychological labor, and physical as well as social separation from shore-based society. These characteristics have been widely discussed in the literature as important factors associated with seafarers' job satisfaction, quality of life, and occupational identity.

Li et al. (24), in their empirical analysis of employment conditions and welfare arrangements among Chinese seafarers, identified key determinants influencing job satisfaction and career continuity. Their result indicate that promotion opportunities exert the strongest effect on job satisfaction, followed by wage levels, welfare benefits, and working conditions. This suggests that seafarers place considerable importance not only on short-term financial compensation but also on long-term career development prospects and institutional stability. Similarly, Tedesco et al. (25) demonstrate that perceived job demands and workload are related to occupational choice and work-related attitudes among seafarers.

The ship as a unique workplace has also been emphasized as a defining feature of the seafaring profession. Kalvaitiene and Sencila (40) conceptualize the vessel not merely as a site of labor but as a social space, highlighting that onboard social relationships and interactions play a crucial role in occupational adjustment and job satisfaction. Furthermore, Tsai and Liou (26) show that motivation, collaborative working environments, and organizational support have a statistically significant impact on seafarers' job performance, underscoring that seafaring is an occupation that depends not only on individual competencies but also on organizational-level support systems. These studies suggest that the distinctive characteristics of seafaring are associated with differences in overall work practices and occupational experiences beyond variations in work arrangements.

### 3.2 Social challenges in the seafaring profession

The occupational distinctiveness of seafaring is accompanied by a range of social challenges, which have contributed to the deterioration of public perceptions of the profession and to the weakening of its long-term sustainability. Previous studies have identified several key social issues facing seafaring, including the undervaluation of occupational status, gender-based barriers to entry, employment insecurity, and mental health problems.

First, the long-standing perception of seafaring as a male-dominated occupation has functioned as a structural barrier limiting women's entry into the profession. Woo (27), based on surveys and interviews with maritime officers, demonstrates that seafaring continues to be embedded in social stereotypes as a “male occupation,” and that such perceptions constrain both occupational choice and career continuity among female maritime professionals. Moreover, the finding that female cadets place greater emphasis on job stability and working conditions suggests that the precarious nature of seafaring employment constitutes a major obstacle to gender diversity within the profession.

Low levels of social recognition relative to the profession's economic and functional contributions also represent a significant social issue confronting seafaring. Choi (28) argues that despite the substantial contribution of Korean seafarers to overseas shipping activities and the national economy, commensurate social recognition and policy support have not been adequately provided. Choi et al. (4), through a comparative analysis of seafarer employment and welfare systems in Korea and China, find that institutional differences may be associated with seafarers' job stability and quality of life. Furthermore, Choi et al. (29) empirically demonstrate that the absence of structured career development programs can lead to a decline in the overall attractiveness of the seafaring profession.

Mental health concerns have also been widely identified as a representative social issue stemming from the structural characteristics of seafaring. Buscema et al. (30) analyze the effects of psychological stressors on job performance and career continuity, showing that psychological wellbeing plays a decisive role in seafarers' job satisfaction. Roberts et al. (31), in their analysis of suicide rates among British merchant seafarers, highlight the adverse impact of prolonged sea service and social isolation on mental health, while Iversen (32) emphasizes that mental health challenges among seafarers remain a persistent structural issue across the global maritime industry.

In this context, Zhang (33) analyses the social problems of Chinese seafarers as structurally rooted in the lack of institutional recognition and social acknowledgment. Chinese seafarers occupy a dual position as both temporary migrant workers and family members, and face prolonged separation from their families, limited social networks, and relative wage disadvantages compared to land-based workers. These conditions have continuously undermined the social status of the seafaring profession. Even during periods when seafarers were regarded as scarce skilled labor, the absence of a robust legal and policy framework to institutionalize their occupational importance and status prevented sustained recognition. Subsequently, amid economic reforms and broader socio-economic transformations, the relative advantages of seafaring further eroded. As a result, seafaring has come to be perceived as an occupation lacking sufficient social recognition and valuation, creating a vicious cycle of declining occupational pride and reduced workforce inflow.

### 3.3 Remark

The existing literature demonstrates that the unique characteristics of the seafaring profession play a significant role in shaping seafarers' professional identity and occupational experiences, while also being associated with various social and structural challenges within the maritime sector. Prolonged periods of sea service, social isolation, the uniqueness of the shipboard working environment, and high job demands distinguish seafaring from other occupations; at the same time, however, these characteristics may contribute to the depreciation of occupational status, gender-based barriers to entry, mental health challenges, and the weakening of long-term career sustainability. Therefore, efforts to improve social perceptions of seafaring must be accompanied by institutional design and policy interventions that appropriately consider the structural and environmental characteristics of maritime occupations.

Based on these findings, the present study developed a multidimensional questionnaire to examine how these occupational characteristics and related social issues are reflected in public perceptions of the seafaring profession. Rather than focusing solely on occupational image, the questionnaire was designed to capture multiple dimensions identified in the literature, including knowledge of the profession, occupational image, career attractiveness, future prospects, perceived social value, working conditions, work–life balance, welfare and remuneration, social recognition, public awareness, and institutional support. These dimensions collectively provide a comprehensive framework for assessing social perceptions of seafaring and their potential relationship with occupational attractiveness and the sustainable development of the maritime workforce.

## 4 Research method

### 4.1 Research design

This study adopts a quantitative research design to examine social perceptions of the seafaring profession from a multidimensional perspective and to empirically identify inter-group differences in perception. The primary objective of the study is to analyze, in a systematic and quantitative manner, perceptual difference regarding the seafaring occupation between the general public and maritime-related groups, including students enrolled in maritime education institutions and active seafarers. To this end, the study employed a structured survey method to collect and analyze data on attitudes and perceptions toward the seafaring profession across multiple population groups. Particular emphasis was placed on accurately capturing overall social perceptions and on conducting a substantive comparison across groups.

The survey was administered to a total of 491 respondents, comprising 204 students (41.5%), 145 members of the general public (29.5%), and 142 active seafarers (28.9%). Given the practical constraints related to physical and social access to the target populations, convenience sampling was adopted as the primary sampling strategy. To enhance sample diversity and coverage, this approach was complemented by snowball sampling. The questionnaire was directly distributed and administered by the researcher through an online platform. Participants were approached by categorizing target groups into students, members of the general public, and active seafarers, after which the completed questionnaires were collected and compiled for analysis. The questionnaire was distributed via an online platform, and data collection was conducted over a 5-month period, from March to August 2025 (see Figure 1).

**Figure 1:**
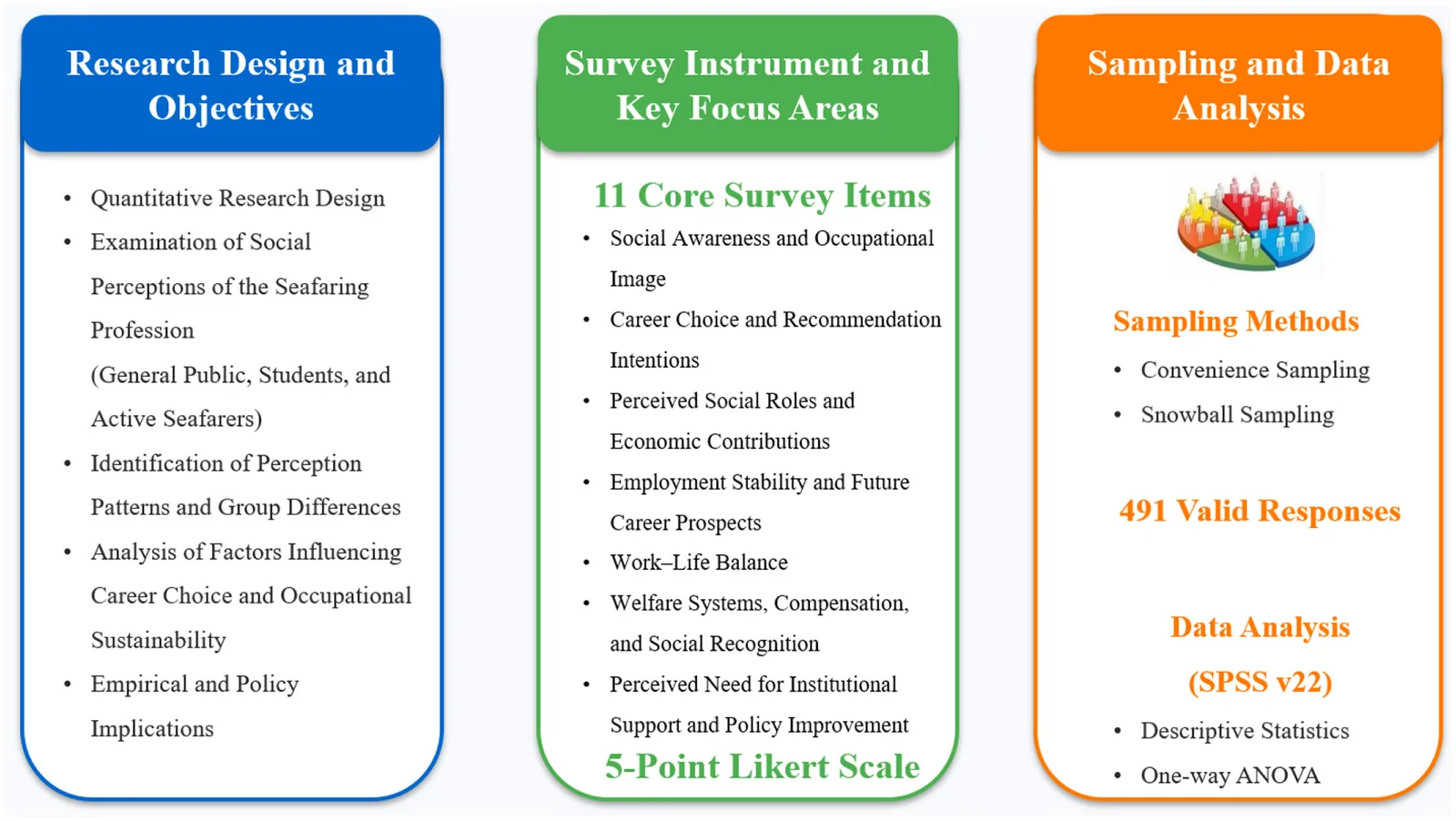
Overview of questionnaire survey.

Survey items were measured using a five-point Likert scale (1 = strongly disagree, 5 = strongly agree), with a value of 3 indicating a neutral perception (34). Scores of 1–2 represent negative perceptions, whereas higher scores indicate a greater level of agreement with each statement (35). In addition, standard deviation was used as an indicator of the degree of dispersion in responses within each group. A smaller standard deviation reflects greater consistency in perceptions among respondents, while a larger value indicates a higher level of divergence. Interpreting mean values in conjunction with standard deviations therefore enables a more comprehensive assessment of both the overall level of perception and the degree of intra-group consensus or heterogeneity.

The questionnaire consisted of 11 items designed to capture multiple dimensions related to perceptions of the seafaring profession, including occupational image, willingness to recommend the profession, employment stability, working conditions, welfare systems, and social recognition. Specifically, the questionnaire comprised eleven items (Q1–Q11), each designed to capture a distinct dimension of social perception related to seafaring. These dimensions include respondents' level of knowledge about the seafaring profession (Q1), perceptions of its occupational image (Q2), willingness to choose the profession or recommend it to others (Q3), perceptions of its future prospects (Q4), perceived importance of seafarers' social roles (Q5), evaluations of working conditions (Q6), perceptions of work–life balance (Q7), assessments of the adequacy of welfare benefits and remuneration (Q8), perceived necessity of improving social recognition of the profession (Q9), the need for public communication and promotion of seafarers' roles and contributions (Q10), and the perceived necessity of institutional and policy support from the government to improve social recognition of the profession (Q11). The data were analyzed using SPSS version 22, employing descriptive statistical techniques to identify overall perceptual tendencies and ANOVA to examine the statistical significance of differences among respondent groups. In addition, ANOVA was performed to examine the statistical significance of inter-group differences. Relevant inferential statistics, including *F*-statistics, degrees of freedom, and *P*-values, were reported for each analysis. Prior to conducting ANOVA, the normality of the score distributions for each group was assessed using the Shapiro–Wilk test (*n* < 50 per cell) and supplemented by inspection of Q–Q plots. All distributions were found to satisfy the normality assumption at the conventional significance level (*P* > 0.05). Homogeneity of variance was evaluated using the *F*-test for each pairwise comparison, and Welch's *t*-test was applied in cases where the equal-variance assumption was violated. In addition, the practical significance of statistically significant ANOVA results was evaluated using eta-squared (η^2^) as a measure of effect size, following conventional benchmarks (small: η^2^ =0.01; moderate: η^2^ =0.06; large: η^2^ =0.14). Effect sizes across the eleven items ranged from small (η^2^ = 0.007 for Q11) to large (η^2^ = 0.150 for Q4), with the largest effects observed for items related to future occupational prospects (Q4: η^2^ = 0.150) and occupational choice and recommendation intentions (Q3: η^2^ = 0.094). Items concerning working conditions and institutional support needs generally yielded small-to-moderate effect sizes (η^2^ ranging from 0.011 to 0.037). The η^2^ values for each item are reported alongside the corresponding ANOVA results in the following subsections, and detailed assumption-testing outcomes are summarized in Appendix A. For survey items showing statistically significant overall group differences, *post hoc* pairwise comparisons were subsequently performed to identify specific between-group differences. Independent two-sample *t*-tests were used for pairwise comparisons, with Welch's *t*-test applied when the assumption of equal variances was violated.

Rather than limiting the analysis to occupational preference or image evaluation alone, this study seeks to systematically elucidate the underlying structure through which social perceptions of the seafaring profession are formed. The study empirically identifies differences in perception across population groups by examining multiple dimensions, including knowledge, image, economic stability, and social significance. In doing so, it provides foundational empirical evidence for understanding the potential for improving social recognition of seafaring and for informing future policy development. Moreover, the study contributes to the literature by offering concrete implications for enhancing the occupational image of seafarers and strengthening societal support for the profession.

However, several potential confounding variables, including age, gender, educational background, prior maritime exposure, and direct affiliation with the maritime sector, were not controlled in the present study. Accordingly, the observed inter-group differences and related findings should be interpreted with caution. It is also necessary to recognize the limitations associated with generalizing the findings beyond the specific sample examined in this study. In particular, the sample cannot be considered representative of the general South Korean population, maritime education students in South Korea as a whole, or the broader population of active seafarers. The use of convenience and snowball sampling may have introduced selection bias, as participants were recruited primarily based on accessibility rather than random selection procedures. Therefore, the observed group differences may partially reflect uncontrolled background characteristics rather than group membership itself.

### 4.2 Reliability and construct validity analysis

The questionnaire used in this study was self-developed by the authors based on previous studies related to occupational perception, seafarer welfare, and maritime workforce sustainability. The questionnaire items were designed to capture multiple dimensions associated with social perceptions of the seafaring profession, including occupational image, career attractiveness, working conditions, welfare systems, and institutional support needs. During the questionnaire development process, conceptually overlapping items were revised and integrated to improve clarity and conceptual consistency.

The final questionnaire consisted of 11 items (Q1–Q11), which were classified into four conceptual dimensions: Dim 1: Social Awareness and Occupational Image (Q1, Q2, Q5), Dim 2: Career Attractiveness (Q3, Q4), Dim 3: Working Conditions and Welfare (Q6, Q7, Q8), and Dim 4: Policy and Institutional Support Needs (Q9, Q10, Q11). To examine the psychometric adequacy of the instrument, reliability and construct validity analyses were conducted. The Cronbach's alpha coefficient for the overall scale was 0.7335, indicating an acceptable level of internal consistency. Subscale analyses further demonstrated acceptable reliability for Dim 1 (α = 0.7153) and Dim 4 (α = 0.7263), while Dim 2 (α = 0.6790) and Dim 3 (α = 0.6843) showed marginal but acceptable reliability levels for exploratory social science research. In addition, the Kaiser–Meyer–Olkin (KMO) measure yielded a value of 0.7787, indicating good sampling adequacy, and Bartlett's test of sphericity was statistically significant (χ^2^_(55)_ = 1,562.903, *P* < 0.001), confirming that the dataset was appropriate for construct validity analysis. Detailed reliability and construct validity results are presented in Appendix B, while the results of assumption testing and effect size analyses for all ANOVA procedures are summarized in Appendix B.

### 4.3 Survey overview

The results of the descriptive statistical analysis of the survey data are presented in Table 3 and Figure 2. With regard to gender distribution, 433 respondents (88.2%) were male and 58 respondents (11.8%) were female. This gender imbalance reflects the structural characteristics of the global maritime industry, in which female seafarers account for approximately 2% of the worldwide seafaring workforce (12). The imbalance is particularly pronounced in South Korea, where the proportion of female maritime professionals remains below the global average. Although women constitute approximately 27.5% of the domestic maritime industry workforce, female officers serving on Korean-flagged vessels account for only 0.7% of all active seafarers (36).

**Table 3:** Descriptive statistics of the sample.

Category	Classification	Frequency (*n*)	Percentage (%)
Gender	Male	433	88.2
	Female	58	11.8
Age	Under 20s	3	0.6
	20s	135	27.5
	30s	123	25.1
	40s	65	13.2
	50s	75	15.3
	60s or above	90	18.3
Education level	Middle school or below	30	6.1
	High school graduate	144	29.3
	University graduate	276	56.2
	Graduate school or above	41	8.4
Target group	Students	204	41.5
	General public	145	29.5
	Seafarers	142	28.9

**Figure 2:**
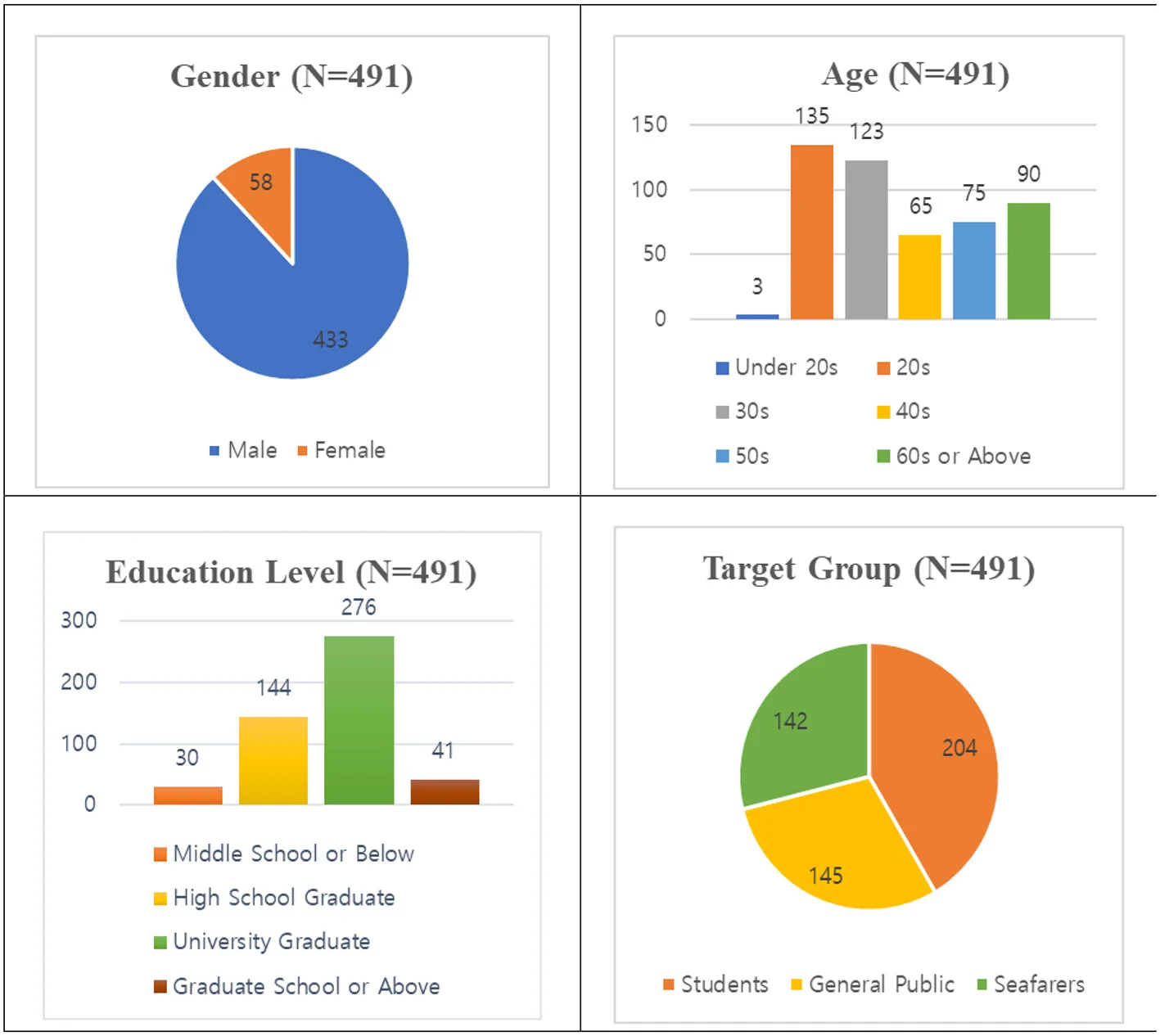
Overview of descriptive statistics of the sample.

In terms of age, three respondents (0.6%) were under 20 years old, 135 respondents (27.5%) were in their 20s, 123 respondents (25.1%) were in their 30s, 65 respondents (13.2%) were in their 40s, 75 respondents (15.3%) were in their 50s, and 90 respondents (18.3%) were aged 60 or older. The largest age group was respondents in their 20s.

Regarding educational attainment, 30 respondents (6.1%) had completed middle school or less, 144 respondents (29.3%) were high school graduates, 276 respondents (56.2%) held a bachelor's degree, and 41 respondents (8.4%) had a postgraduate degree or higher. The majority of respondents were university graduates.

With respect to respondent groups, students accounted for the largest proportion with 204 respondents (41.5%), followed by the general public with 145 respondents (29.5%) and active seafarers with 142 respondents (28.9%).

### 4.4 Survey questions on social perceptions of the seafaring profession

#### 4.4.1 Knowledge of the seafaring profession (Q1)

To examine differences in perceived knowledge regarding the question “How well do you know the seafaring profession?” ANOVA was conducted across three groups: students, the general public, and active seafarers. As shown in Table 4, the descriptive statistics indicate that the student group reported a mean score of 3.49 (SD = 0.5664), the general public group a mean of 3.59 (SD = 0.7165), and the seafarer group a mean of 4.06 (SD = 0.7123). These results demonstrate that seafarers exhibit the highest level of self-reported knowledge of the profession, while the student and general public groups show relatively lower levels.

**Table 4:** Results of descriptive statistics for knowledge of the seafaring profession (Q1).

Group	Mean	Standard deviation
Students	3.4902	0.5664
General public	3.5862	0.7165
Seafarers	4.0634	0.7123

According to Table 5, the results of y ANOVA showed that the between-group sum of squares was 29.49 (df = 2) with a mean square of 14.74, while the within-group sum of squares was 318.58 (df = 488). The analysis revealed a statistically significant difference among the groups (*F*_(2,488)_ = 22.584, *P* < 0.001).

**Table 5:** Results of ANOVA for Q1.

Source of variation	SS	df	MS	*F*	*P*-value	*F* crit
Between groups	29.487	2	14.743	22.584	0.000	3.0142
Within groups	318.58	488	0.6528			
Total	348.07	490				

Independent two-sample *t*-tests assuming equal variances were conducted as *post hoc* analyses, as the assumption of homogeneity of variance was satisfied (Table 6). The results implies that active seafarers demonstrated comparatively higher levels of occupational knowledge and familiarity with the seafaring profession than students and members of the general public.

**Table 6:** Results of *F*-test and *t*-test among groups for Q1.

Groups	*F*-test			*T*-test		
	*F*-ratio	*P*-value	Variance hypothesis	*t*-statistic	*P*-value	Mean
Student-Public	0.7905	6.2	Equal variance	−1.115	13	Same
Public-Seafarer	1.0059	48.6	Equal variance	−4.782	0.00	Different
Student-Seafarer	0.7952	6.8	Equal variance	−6.628	0.00	Different

#### 4.4.2 Occupational image (Q2)

According to the analysis of responses to the question, “What is your perception of the image of the seafaring profession?” the student group recorded the highest mean score at 3.25 (SD = 0.88), followed by the seafarer group with a mean of 3.01 (SD = 0.84), and the general public group with a mean of 2.91 (SD = 0.92; See Table 7). These results indicate that students hold a relatively more positive image of the seafaring profession than the other groups. Notably, individuals with no direct occupational experience (students) exhibited a more favorable perception than those currently engaged in the profession.

**Table 7:** Results of descriptive statistics for occupational image (Q2).

Group	Mean	Standard deviation
Students	3.2450	0.8755
General public	2.9103	0.9155
Seafarers	3.0070	0.8439

As presented in Table 8, the between-group sum of squares was 10.4865 (df = 2) with a corresponding mean square of 5.2432, whereas the within-group sum of squares was 428.5725 (df = 488). The ANOVA results demonstrated statistically significant inter-group differences in perceptions of occupational image (*F*_(2,488)_ = 5.9703, *P* = 0.002).

**Table 8:** Results of ANOVA for Q2.

Source of variation	SS	df	MS	*F*	*P*-value	*F* crit
Between groups	10.4865	2	5.2432	5.9703	0.002	3.0141
Within groups	428.5725	488	0.8782			
Total	439.0591	490				

The *post hoc* analyses further showed that students perceived the occupational image of the seafaring profession more positively than both the general public and active seafarers, whereas perceptions between the general public and seafarers appeared relatively similar (Table 9).

**Table 9:** Results of *F*-test and *t*-test among groups for Q2.

Groups	*F*-test			*T*-test		
	*F*-ratio	*P*-value	Variance hypothesis	*t*-statistic	*P*-value	Mean
Student-Public	0.9563	38.3	Equal variance	3.2627	0.1	Different
Public-Seafarer	1.0848	31.4	Equal variance	−0.8730	19.2	Same
Student-Seafarer	1.0375	41.0	Equal variance	2.3452	1.0	Different

#### 4.4.3 Occupational choice and willingness to recommend (Q3)

Responses to the question, “Would you choose the occupation of a seafarer yourself or recommend it to people close to you?” were compared across the three groups. Table 10 indicate that the student group recorded the highest mean score at 3.59 (SD = 0.92), followed by the general public group with a mean of 3.21 (SD = 0.99). In contrast, the seafarer group reported the lowest mean score at 2.84 (SD = 0.95). While students demonstrated the strongest intention to choose or recommend the occupation, seafarers showed the weakest inclination, suggesting notable differences in attitudes and expectations toward the occupation across groups. The descriptive statistical analysis reveals clear group-based differences in perceptions regarding the intention to choose or recommend the seafaring profession.

**Table 10:** Results of descriptive statistics for occupational choice and willingness to recommend (Q3).

Group	Mean	Standard deviation
Students	3.5931	0.9223
General public	3.2068	0.9985
Seafarers	2.8380	0.9452

The ANOVA results demonstrated statistically significant inter-group differences in willingness to choose and recommend the seafaring profession (*F*_(2,488)_ = 25.400, *P* < 0.001). The between-group sum of squares was 48.3332 (df = 2) with a corresponding mean square of 24.166, whereas the within-group sum of squares was 464.2981 (df = 488; See Table 11).

**Table 11:** Results of ANOVA for Q3.

Source of variation	SS	df	MS	*F*	*P*-value	*F* crit
Between groups	48.3332	2	24.166	25.4002	0.001	3.0141
Within groups	464.2981	488	0.9514			
Total	512.6314	490				

In Table 12, the *post hoc* comparison results for Q3 indicated significant differences in willingness to choose and recommend the seafaring profession varied significantly across the three groups, with the largest perceptual gap observed between students and seafarers.

**Table 12:** Results of *F*-test and *t*-test among groups for Q3.

Groups	*F*-test			*T*-test		
	*F*-ratio	*P*-value	Variance hypothesis	*t*-statistic	*P*-value	Mean
Student-Public	0.9236	30.0	Equal variance	3.6406	0.0	Different
Public-Seafarer	1.0564	37.2	Equal variance	3.1687	0.1	Different
Student-Seafarer	0.9757	43.4	Equal variance	7.1580	0.0	Different

#### 4.4.4 Future prospects of the seafaring occupation (Q4)

Perceptions of the future prospects of the seafaring occupation were analyzed across three groups: students, the general public, and active seafarers. Table 13 indicate that the student group reported a mean score of 3.62 (SD = 0.82), reflecting a generally positive outlook. The general public group showed a moderately positive perception, with a mean score of 3.39 (SD = 0.85). In contrast, the seafarer group recorded a mean score of 2.72 (SD = 0.79), the only group with a score below the neutral midpoint, indicating a relatively negative perception of future prospects.

**Table 13:** Results of descriptive statistics for future prospects of the seafaring occupation (Q4).

Group	Mean	Standard deviation
Students	3.6225	0.8174
General public	3.3931	0.8513
Seafarers	2.7183	0.7853

The between-group sum of squares was 70.5060 (df = 2) with a mean square of 35.2530, while the within-group sum of squares was 399.2618 (df = 488). The analysis revealed a statistically significant difference among the groups in perceptions of future prospects (*F*_(2,488)_ = 43.0882, *P* < 0.001; See Table 14).

**Table 14:** Results of ANOVA for Q4.

Source of variation	SS	df	MS	*F*	*P*-value	*F* crit
Between groups	70.5060	2	35.2530	43.0882	0.001	3.0141
Within groups	399.2618	488	0.8181			
Total	469.7678	490				

The three groups demonstrated significantly different perceptions regarding the future prospects of the seafaring profession, with the seafarer group being clearly distinguished from the other groups.

#### 4.4.5 Perceived social role of seafarers (Q5)

For the item measuring the perception of whether seafarers play an important role in society, the means and variances of the student, general public, and seafarer groups were compared. The results indicate that the student group recorded a mean score of 4.23 (SD = 0.621), the general public group 4.02 (SD = 0.527), and the seafarer group 3.85 (SD = 0.731; See Table 15). All three groups demonstrated a generally positive attitude toward the statement that seafarers play an important social role.

**Table 15:** Results of descriptive statistics for perceived social role of seafarers (Q5).

Group	Mean	Standard deviation
Students	4.2303	0.6215
General public	4.0275	0.5270
Seafarers	3.8591	0.7317

Based on Table 16, the ANOVA results indicated statistically significant inter-group differences in perceptions regarding the social role of seafarers (*F*_(2,488)_ = 9.4312, *P* < 0.001). The between-group sum of squares was 11.7984 (df = 2) with a corresponding mean square of 5.8992, whereas the within-group sum of squares was 305.2443 (df = 488).

**Table 16:** Results of ANOVA for Q5.

Source of variation	SS	df	MS	*F*	*P*-value	*F* crit
Between groups	11.7984	2	5.8992	9.4312	0.001	3.0141
Within groups	305.2443	488	0.6255			
Total	317.0428	490				

In Table 17, the three groups differed significantly in their perceptions of the social role of seafarers, suggesting the need for group-specific strategies to improve social awareness.

**Table 17:** Results of *F*-test and *t*-test among groups for Q5.

Groups	*F*-test			*T*-test		
	*F*-ratio	*P*-value	Variance hypothesis	*t*-statistic	*P*-value	Mean
Student-Public	1.1793	14.6	Equal variance	2.447	0.7	Different
Public-Seafarer	0.7201	2.5	Unequal variance	1.797	3.7	Different
Student-Seafarer	0.8493	14.3	Equal variance	4.160	0.0	Different

### 4.5 Questions on the working environment and welfare of the seafaring occupation

#### 4.5.1 Working environment of the seafaring occupation (Q6)

Perceptions of the working environment of seafarers were analyzed across three groups: students, the general public, and seafarers. Table 18 presents the results, showing that the mean score for the student group was 2.75 (SD = 0.90), for the general public group 2.89 (SD = 0.90), and for the seafarer group 2.46 (SD = 0.79). All three mean values fell below 3.0 (“neutral”), suggesting that respondents across groups share an overall negative perception of the working environment in the seafaring occupation.

**Table 18:** Results of *F*-test and *t*-test among Groups for Q6.

Groups	*F*-test			*T*-test		
	*F*-ratio	*P*-value	Variance hypothesis	*t*-statistic	*P*-value	Mean
Student-Public	0.9899	47.0	Equal variance	−1.3083	9.6	Same
Public-Seafarer	1.1462	20.8	Equal variance	3.9742	0.0	Different
Student-Seafarer	1.1462	20.8	Equal variance	2.9461	0.2	Different

Perceptions of the working environment differed significantly across the three groups according to the ANOVA findings (*F*_(2,488)_ = 8.1441, *P* < 0.001). The analysis produced a between-group sum of squares of 14.1262 with 2 degrees of freedom and a mean square of 7.0631, whereas the within-group variation accounted for a sum of squares of 423.2261 with 488 degrees of freedom.

*Post hoc* comparisons for Q6 indicate that perceptions of the working environment vary across groups, with the seafarer group being distinctly differentiated from the other groups, highlighting the need for policy and educational interventions tailored to group characteristics.

#### 4.5.2 Work–life balance (Q7)

Perceptions of whether the seafaring profession supports the maintenance of work–life balance were analyzed across three groups: students, the general public, and active seafarers. According to the data in Table 19, the mean score for the student group was 2.83 (SD = 0.94), that for the general public was 2.48 (SD = 0.72), and that for the seafarer group was 2.86 (SD = 0.69). All three group means fell below the neutral midpoint of 3 (“neither agree nor disagree”), suggesting a generally weak perception that seafaring is an occupation conducive to maintaining an adequate work–life balance. Notably, the general public recorded the lowest mean score (2.48), while the student and seafarer groups reported similar average levels 2.83 and 2.86, respectively.

**Table 19:** Results of descriptive statistics for work–life balance (Q7).

Group	Mean	Standard deviation
Students	2.8333	0.9376
General public	2.4758	0.7233
Seafarers	2.8591	0.6892

Table 20 shows that perceptions of work–life balance varied significantly among the respondent groups. The ANOVA produced an *F*-value of 8.6581 with a significance level of *P* = 0.0002 (*F*_(2,488)_ = 8.6581, *P* = 0.0002). The between-group variation accounted for a sum of squares of 13.8985 (df = 2) and a mean square of 6.9492, whereas the within-group sum of squares was calculated as 391.6819 (df = 488).

**Table 20:** Results of ANOVA for Q7.

Source of variation	SS	df	MS	*F*	*P*-value	*F* crit
Between groups	13.8985	2	6.9492	8.6581	0.0002	3.0141
Within groups	391.6819	488	0.8026			
Total	405.5804	490				

Pairwise comparisons were performed using independent two-sample *t*-tests after assessing the assumption of homogeneity of variance. These results suggest that perceptions of work–life balance varied across respondent groups, with the general public demonstrating perceptual characteristics distinct from those of both students and seafarers.

#### 4.5.3 Adequacy of welfare and salary (Q8)

Perceptions of whether the level of welfare benefits and remuneration for seafarers is adequate were analyzed across three groups: students, the general public, and seafarers. The descriptive statistics show that the mean score for the student group was 2.70 (SD = 0.91), for the general public 2.66 (SD = 0.78), and for the seafarer group 2.48 (SD = 0.79; See Table 21). All three groups reported mean values below 3 (“neutral”), indicating a shared perception that welfare and compensation for seafarers are insufficient rather than adequate.

**Table 21:** Results of descriptive statistics for adequacy of welfare and salary (Q8).

Group	Mean	Standard deviation
Students	2.7009	0.9101
General public	2.6620	0.7808
Seafarers	2.4788	0.7903

The between-group sum of squares was 4.4090 (df = 2) with a mean square of 2.2045, while the within-group sum of squares was 408.6378 (df = 488). The resulting *F*-value was 2.6326 (*P* < 0.0729), indicating that overall group differences in perceptions of the adequacy of welfare and remuneration were not statistically significant.

Because the omnibus ANOVA result was not statistically significant, no *post hoc* pairwise comparisons were conducted for Q8 (See Table 22).

**Table 22:** Results of *F*-test and *t*-test among groups for Q8.

Groups	*F*-test			*T*-test		
	*F*-ratio	*P*-value	Variance hypothesis	*t*-statistic	*P*-value	Mean
Student-Public	1.1655	16.4	Equal variance	0.3870	34.9	Same
Public-Seafarer	0.9879	47.1	Equal variance	1.7507	4.1	Different
Student-Seafarer	1.1516	18.5	Equal variance	2.1901	1.5	Different

### 4.6 Question on the need for institutional measures to improve social perceptions of the seafaring profession

#### 4.6.1 Necessity of improving social perceptions (Q9)

Perceptions regarding the need to improve the social recognition of seafarers were analyzed. Based on Table 23, The descriptive statistics indicate that the student group recorded a mean score of 3.88 (standard deviation = 0.91), the general public group a mean of 3.66 (standard deviation = 0.84), and the seafarer group a mean of 4.08 (standard deviation = 0.67). All three groups reported mean values above 3.5, suggesting a broad consensus that improvements in the social recognition of seafarers are necessary.

**Table 23:** Results of descriptive statistics for necessity of improving social perceptions (Q9).

Group	Mean	Standard deviation
Students	3.8823	0.9121
General public	3.6551	0.8386
Seafarers	4.0845	0.6736

The seafarer group exhibited the highest mean score (4.08), reflecting their direct experience of the limitations, misunderstandings, or lack of recognition associated with the occupation. Both the general public and student groups also demonstrated relatively high mean values exceeding 3.5, indicating that external groups likewise perceive that the seafaring profession is not sufficiently recognized or valued in society.

The ANOVA results indicated that the between-group sum of squares was 13.2500 (df = 2) with a mean square of 6.6250, while the within-group sum of squares was 400.9210 (df = 488), yielding a total sum of squares of 414.1711 (df = 490). The calculated *F*-value was 8.0639 with a significance level of *P* =0.0003 (*F*_(2,488)_ = 8.0639, *P* 0.0003), indicating statistically significant differences among the groups (Table 24). These results presume that the mean perceptions regarding the corresponding survey item differed significantly across the three groups, thereby necessitating *post hoc* analyses to identify the specific group pairs in which the differences occurred.

**Table 24:** Results of ANOVA for Q9.

Source of variation	SS	df	MS	*F*	*P*-value	*F* crit
Between groups	13.2500	2	6.6250	8.0639	0.0003	3.0141
Within groups	400.9210	488	0.8215			
Total	414.1711	490				

*Post hoc* comparisons for Q9 indicated that active seafarers expressed comparatively stronger perceptions regarding the necessity of improving social recognition of the seafaring profession than students and members of the general public. In addition, Welch's *t*-test was applied to the student–seafarer comparison due to unequal variance assumptions (Table 25).

**Table 25:** Results of *F*-test and *t*-test among groups for Q9.

Groups	*F*-test			*T*-test		
	*F*-ratio	*P*-value	Variance hypothesis	*t*-statistic	*P*-value	Mean
Student-Public	1.0877	29.6	Equal variance	2.2274	1.3	Different
Public-Seafarer	1.2448	9.6	Equal variance	−4.1796	0.0	Different
Student-Seafarer	1.3540	2.7	Unequal variance	−2.10581	1.8	Different

#### 4.6.2 Necessity of promotional activities (Q10)

A survey addressing the statement, “Do you think promotional activities are necessary to raise public awareness of the role and importance of seafarers?” was conducted among three groups: students, the general public, and seafarers. Table 26 indicated mean scores of 4.18 (SD = 0.50) for students, 4.08 (SD = 0.42) for the general public, and 3.97 (SD = 0.69) for seafarers. All groups exhibited mean scores near 4, reflecting a high level of agreement and suggesting broad societal recognition of the need for promotional activities to enhance public understanding of seafarers' roles and importance.

**Table 26:** Results of descriptive statistics for necessity of promotional activities (Q10).

Group	Mean	Standard deviation
Students	4.1813	0.5038
General public	4.0758	0.4178
Seafarers	3.9718	0.6942

In Table 27, the ANOVA results demonstrated statistically significant differences among the groups in perceptions regarding the necessity of promotional activities (*F*_(2,488)_ = 3.4817, *P* = 0.0315). The between-group sum of squares was 3.7149 (df = 2) with a corresponding mean square of 1.8574, while the within-group sum of squares was 260.3421 (df = 488).

**Table 27:** Results of ANOVA for Q10.

Source of variation	SS	df	MS	*F*	*P*-value	*F* crit
Between groups	3.7149	2	1.8574	3.4817	0.0315	3.0141
Within groups	260.3421	488	0.5334			
Total	264.0570	490				

*Post hoc* analyses following the ANOVA were conducted using *F*-tests and independent two-sample *t*-tests to further examine inter-group differences in perceptions regarding the need for promotional activities (Table 28). These results may mean that perceptions concerning the necessity of promotional activities varied across respondent groups, with the most distinct difference identified between students and active seafarers.

**Table 28:** Results of *F*-test and *t*-test among groups for Q10.

Groups	*F*-test			*T*-test		
	*F*-ratio	*P*-value	Variance hypothesis	*t*-statistic	*P*-value	Mean
Student-Public	1.206	11.5	Equal variance	1.4196	7.8	Same
Public-Seafarer	0.602	0.1	Unequal variance	1.1801	11.9	Same
Student-Seafarer	0.726	1.8	Unequal variance	2.4426	0.8	Different

#### 4.6.3 Necessity of institutional support from the government (Q11)

A survey on the perceived necessity of governmental support for improving public awareness of seafarers was conducted among students, the general public, and seafarers. Descriptive statistics from Table 29 reveal that mean scores of 4.33 (SD = 0.47) for students, 4.20 (SD = 0.47) for the general public, and 4.23 (SD = 0.63) for seafarers. All groups scored well-above 4, reflecting strong agreement on the need for governmental institutional support to enhance social recognition of the seafaring profession.

**Table 29:** Results of descriptive statistics for necessity of institutional support from the government (Q11).

Group	Mean	Standard deviation
Students	4.3333	0.4696
General public	4.2000	0.4666
Seafarers	4.2323	0.6335

Table 30 showed that perceptions regarding the necessity of governmental institutional support did not significantly differ among the groups according to the ANOVA results (*F*_(2,488)_ = 1.6625, *P* =0.1907). The between-group sum of squares was 1.7161 (df = 2) with a corresponding mean square of 0.8580, whereas the within-group sum of squares was 251.8643 (df = 488).

**Table 30:** Results of ANOVA for Q11.

Source of variation	SS	df	MS	*F*	*P*-value	*F* crit
Between groups	1.7161	2	0.8580	1.6625	0.1907	3.0141
Within groups	251.8643	488	0.5161			
Total	253.5804	490				

*Post hoc* comparisons for Q11 revealed a statistically significant difference only between students and members of the general public regarding perceptions of the necessity of governmental institutional support for the seafaring profession. By contrast, the remaining pairwise comparisons did not demonstrate statistically significant differences (Table 31).

**Table 31:** Results of *F*-test and *t*-test among groups for Q11.

Groups	*F*-test			*T*-test		
	*F*-ratio	*P*-value	Variance hypothesis	*t*-statistic	*P*-value	Mean
Student-Public	1.0063	48.7	Equal variance	1.7935	3.7	Different
Public-Seafarer	0.7365	3.4	Unequal variance	−0.3696	35.6	Same
Student-Seafarer	0.7412	2.5	Unequal variance	1.2273	11.0	Same

## 5 Discussion

This study identified significant differences in social perceptions of the seafaring profession among students, members of the general public, and active seafarers. As shown in Tables 10–14, 32, students reported the most positive perceptions regarding occupational attractiveness, willingness to recommend the profession, and future career prospects, whereas active seafarers consistently expressed less favorable views. In particular, the largest inter-group differences were observed for occupational choice and recommendation intentions (Q3) and perceptions of future prospects (Q4), indicating that direct occupational experience is closely associated with evaluations of the profession.

**Table 32:** Results of *F*-test and *t*-test among groups for Q4.

Groups	*F*-test			*T*-test		
	*F*-ratio	*P*-value	Variance hypothesis	*t*-statistic	*P*-value	Mean
Student-Public	0.9601	39.3	Equal variance	2.3165	1.06	Different
Public-Seafarer	1.0840	31.6	Equal variance	6.3168	0.0	Different
Student-Seafarer	1.0408	40.2	Equal variance	9.2257	0.0	Different

These findings are consistent with previous studies emphasizing that occupational attractiveness among seafarers is strongly related to working conditions, welfare systems, career development opportunities, and institutional support (4, 24, 29). While students and members of the general public tended to view seafaring as a socially valuable and attractive occupation, active seafarers reported substantially more critical assessments. This discrepancy suggests that external perceptions of the profession do not fully reflect the realities experienced within maritime workplaces.

The results related to working conditions and welfare further reinforce this interpretation. As shown in Tables 18–22, 33–36, all groups evaluated working conditions, work–life balance, and welfare provisions relatively negatively, with active seafarers reporting the lowest scores in most categories. Previous research has similarly shown that prolonged sea service, occupational isolation, heavy workloads, and limited welfare support are important challenges affecting seafarers' occupational experiences and job satisfaction (24, 25). The present findings extend this literature by demonstrating that these concerns are reflected not only in occupational experiences but also in broader perceptions of career sustainability and occupational attractiveness.

**Table 33:** Results of descriptive statistics for working environment of the seafaring occupation (Q6).

Group	Mean	Standard deviation
Students	2.7549	0.8952
General public	2.8896	0.9044
Seafarers	2.4577	0.7889

**Table 34:** Results of ANOVA for Q6.

Source of variation	SS	df	MS	*F*	*P*-value	*F* crit
Between groups	14.1262	2	7.0631	8.1441	0.0003	3.0141
Within groups	423.2261	488	0.8672			
Total	437.3523	490				

**Table 35:** Results of *F*-test and *t*-test among groups for Q7.

Groups	*F*-test			*T*-test		
	*F*-ratio	*P*-value	Variance hypothesis	*t*-statistic	*P*-value	Mean
Student-Public	1.2961	4.9	Unequal variance	3.6513	0.0	Different
Public-Seafarer	1.0495	38.7	Equal variance	−3.8624	0.0	Different
Student-Seafarer	1.3603	2.6	Unequal variance	−0.2656	39.5	Same

**Table 36:** Results of ANOVA for Q8.

Source of variation	SS	df	MS	*F*	*P*-value	*F* crit
Between groups	4.4090	2	2.2045	2.6326	0.0729	3.0141
Within groups	408.6378	488	0.8373			
Total	413.0648	490				

Another important implication is that all respondent groups recognized the need to improve social recognition of the seafaring profession. Although perceptions differed regarding occupational attractiveness and working conditions, respondents broadly agreed that seafarers perform socially important roles and that greater institutional support is required. This finding supports earlier studies arguing that insufficient social recognition and limited institutional support undermine workforce recruitment and long-term career continuity within the maritime sector (22, 33). The results therefore suggest that workforce sustainability cannot be addressed through public promotion alone. Efforts to improve the public image of seafaring should be accompanied by tangible improvements in working environments, welfare systems, employment stability, and career development opportunities.

Such results indicate that social perceptions of the seafaring profession are shaped by differences in occupational experience, professional exposure, and institutional conditions. They also highlight the importance of addressing both perceptual and structural factors when developing policies aimed at strengthening the attractiveness and long-term sustainability of the maritime workforce.

Based on the results of this study, several practical implications emerge for governments, maritime education institutions, and shipping companies. Improving public perceptions of the seafaring profession requires not only public awareness campaigns but also policies that enhance employment stability, welfare systems, and long-term career development to address the structural factors underlying negative perceptions of the profession. Maritime education institutions should provide prospective students with balanced and realistic information regarding both the opportunities and challenges of seafaring careers while strengthening career guidance and collaboration with the shipping industry to support informed career decisions. Shipping companies should also place greater emphasis on improving onboard working conditions, work–life balance, welfare benefits, and career development systems rather than focusing solely on recruitment, as these factors are closely associated with occupational attractiveness and long-term workforce retention. Collectively, these policy and institutional efforts may contribute to enhancing public recognition of the seafaring profession and supporting the sustainable development of the maritime workforce.

## 6 Conclusion

This study empirically examined social perceptions of the seafaring profession by comparing students, members of the general public, and active seafarers. The findings revealed significant differences across groups, with active seafarers generally expressing less favorable perceptions of working conditions, welfare systems, and long-term career sustainability than external groups. At the same time, respondents across all groups recognized the importance of improving social recognition and institutional support for the seafaring profession.

These findings have broader implications for the sustainability of the maritime workforce. While enhancing public awareness and the social image of seafaring remains important, the results suggest that long-term workforce sustainability is also closely related to the quality of occupational conditions experienced by seafarers. Efforts to strengthen the attractiveness of the profession should therefore extend beyond promotional initiatives and include improvements in working conditions, welfare support, employment stability, and career development opportunities.

The practical significance of this study lies in providing empirical evidence that may assist policymakers, maritime education institutions, shipping companies, and other industry stakeholders in developing more effective workforce strategies. Future research should build on these findings by employing more representative sampling approaches and examining how social perceptions of seafaring evolve across different demographic, occupational, and national contexts. Such research would contribute to a deeper understanding of workforce attraction, retention, and sustainability within the maritime sector.

Future research should employ more representative, probability-based sampling methods to enhance the representativeness and external validity of the findings. In addition, cross-national comparative studies are needed to investigate how social perceptions of the seafaring profession are shaped and evolve across different demographic, occupational, and national contexts. Such research would improve the generalizability of the findings while providing a more comprehensive understanding of the factors influencing workforce attraction, retention, and the sustainable development of the maritime workforce.

## Data Availability

The original contributions presented in the study are included in the article/Supplementary material, further inquiries can be directed to the corresponding author.
