# Supplementary material for: Social perceptions of seafarers and the sustainable development of the maritime workforce: a questionnaire survey in South Korea
Source: Front Public Health. 2026 Sep 21;14:1927794. doi: 10.3389/fpubh.2026.1927794 (PMC13637671; doi:10.3389/fpubh.2026.1927794)
Supplement: Supplementary file 1 [file Supplementary_file_1.docx]

Appendix A. Assumption Testing and Effect Size Results

| Item | Normality | Homogeneity of Variance | Effect Size (η²) | Interpretation |
| --- | --- | --- | --- | --- |
| Q1 Knowledge of the Seafaring Profession | Satisfied | Satisfied | 0.085 | Moderate |
| Q2 Occupational Image of the Seafaring Profession | Satisfied | Satisfied | 0.024 | Small |
| Q3 Occupational Choice and Willingness to Recommend | Satisfied | Satisfied | 0.094 | Moderate |
| Q4 Future Prospects of the Seafaring Occupation | Satisfied | Satisfied | 0.150 | Large |
| Q5 Perceived Social Role of Seafarers | Satisfied | Satisfied | 0.037 | Small–Moderate |
| Q6 Working Environment of the Seafaring Occupation | Satisfied | Satisfied | 0.032 | Small–Moderate |
| Q7 Work–Life Balance | Satisfied | Partially Satisfied* | 0.034 | Small–Moderate |
| Q8 Adequacy of Welfare and Salary | Satisfied | Satisfied | 0.011 | Small |
| Q9 Necessity of Improving Social Perceptions | Satisfied | Partially Satisfied** | 0.032 | Small–Moderate |
| Q10 Necessity of Promotional Activities | Satisfied | Satisfied | 0.014 | Small |
| Q11 Necessity of Institutional Support from the Government | Satisfied | Partially Satisfied*** | 0.007 | Small |

Note. Effect size interpretation followed conventional η² criteria: small = .01, moderate = .06, and large = .14.

* Unequal variance assumptions were applied for some pairwise comparisons in the post hoc analysis.
** Welch’s t-test was applied for the student–seafarer comparison due to unequal variance assumptions.
*** Unequal variance assumptions were identified in several pairwise comparisons involving the seafarer group.

Appendix B. Reliability and Construct Validity Analysis Results

Cronbach’s Alpha Results for Overall Scale and Subscales

| **Scale / Subscale** | **Items** | **α** | **Interpretation** |
| --- | --- | --- | --- |
| Overall scale (k = 11) | Q1–Q11 | 0.7335 | Acceptable (≥ 0.70) |
| Dim 1 — Social Awareness & Image | Q1, Q2, Q5 | 0.7153 | Acceptable (≥ 0.70) |
| Dim 2 — Career Attractiveness | Q3, Q4 | 0.6790 | Marginal (0.60–0.69) |
| Dim 3 — Working Conditions & Welfare | Q6, Q7, Q8 | 0.6843 | Marginal (0.60–0.69) |
| Dim 4 — Policy & Institutional Need | Q9, Q10, Q11 | 0.7263 | Acceptable (≥ 0.70) |

*Note. Criteria: α ≥ .70 = acceptable; α .60–.69 = marginal; α < .60 = low (Nunnally & Bernstein, 1994).*

Table Construct Validity Preconditions — KMO & Bartlett's Test

| **Test** | **Value** | **Interpretation** |
| --- | --- | --- |
| KMO (overall) | 0.7787 | ✓ Good (≥ 0.70) — Factor analysis is appropriate |
| Bartlett's χ²(55) | 1562.903 |  |
| Bartlett's p-value | < .001 | ✓ Significant — Items are sufficiently correlated |
